# ATG5-FOXA3 Axis Contributes to Lysosomal Biogenesis and Auditory Function in Kölliker’s Organ

**DOI:** 10.3390/biomedicines14040802

**Published:** 2026-04-01

**Authors:** Penghui Chen, Jifang Zhang, Ying Wang, Jiarui Chen

**Affiliations:** 1 Department of Otolaryngology-Head and Neck Surgery, Shanghai Children’s Hospital, Shanghai Jiaotong University School of Medicine, Shanghai 200127, China; cph_shsmu@163.com; 2 Department of Otorhinolaryngology-Head and Neck Surgery, Xinhua Hospital, Shanghai Jiaotong University School of Medicine, Shanghai 200082, China; jifang_zhang@outlook.com

**Keywords:** Kölliker’s organ, FOXA3, ATG5, lysosome, auditory development

## Abstract

Background: Kölliker’s organ (KO) support cells undergo orderly, time-dependent degeneration that is essential for auditory development and is accompanied by precisely regulated autophagic activity; however, the molecular hierarchy linking autophagy to this remodeling remains obscure. This study aimed to elucidate the regulatory mechanisms connecting autophagic flux to lysosomal biogenesis and auditory function during cochlear development. Method: We established an *Atg5^flox/flox^; Sox2Cre+* mouse model with deletion of the autophagy gene Atg5 in cochlear-supporting cells. Auditory function was assessed via Auditory Brainstem Response (ABR) testing. Transcriptomic profiling of the neonatal basilar membrane was performed to screen for downstream targets. Mechanistic validation included spatiotemporal immunofluorescence mapping (E18–P30) and in vitro functional assays using siRNA-mediated knockdown and lysosomal tracking. Results: At 2 months of age, *Atg5^flox/flox^; Sox2Cre+* mice exhibited moderate-to-severe sensorineural hearing loss accompanied by significant outer hair cell loss. Bulk RNA-seq of the basilar membrane identified fork-head box A3 (*Foxa3*) as a significantly downregulated transcription factor within the lysosomal–autophagy network. Spatiotemporal immunolabelling from embryonic day 18 to postnatal day 30 revealed that FOXA3 expression becomes progressively restricted to KO cells during postnatal development, with ATG5 loss reducing FOXA3 protein levels by 62.4%. In vitro, deficiency of either *Atg5* or *Foxa3* in primary KO cells resulted in comparable reductions in LAMP1-positive puncta. Conclusions: These findings support a model wherein the ATG5-FOXA3 axis contributes to lysosomal biogenesis in developing KO cells, with implications for understanding mechanisms of congenital sensorineural hearing loss.

## 1. Introduction

Multicellular organism development demands precise coordination of cellular events like proliferation, death, and differentiation [1]. Cells acquire energy or form new components by eliminating or recycling their own parts [2]. After birth, the mammalian auditory system experiences morphological and gene expression changes, with the cochlear Kölliker’s organ (KO) being a pivotal structure [3]. As a supporting cell group near the cochlear axis medial to inner hair cells, the KO is crucial for auditory development, promoting tectorial membrane development in the organ of Corti, serving as a source for inner hair cell differentiation, and facilitating hair cell maturation and auditory acquisition via ATP release [4,5,6,7]. However, prior to auditory acquisition, KO supporting cells degenerate from the medial to lateral and basal to apical regions, forming the inner sulcus. Delayed or impaired KO degeneration can disrupt the organ of Corti’s development, causing auditory impairment [8]. Thus, studying KO degeneration mechanisms is vital for hearing loss research and intervention.

Our prior work revealed that during neonatal rat cochlear development, KO-support cell numbers decline and morphology changes. These cells undergo both apoptosis and proliferation in vivo, but proliferation is far less extensive than apoptosis [9]. The expression of KO apoptosis-related factors in postnatal rats is time-dependent, peaking at postnatal day 3–5 (P3–5) [10], and another study set the apoptosis window at P7–P13 [9]. Apoptosis is significant in KO degeneration. Apoptosis-deficient mice show delayed KO regression and severe hearing loss, yet it cannot explain the massive cell loss before P5 [8]. We also found autophagy during KO supporting cell degeneration, with autophagic flux linked to the degeneration process. Autophagy and apoptosis may have distinct roles at different developmental stages [11].

Lysosome biogenesis and autophagy are mutually regulated [12]. Early in development, lysosomes are highly dynamic, maintaining function through fusion and fission and interacting with other organelles [13]. Lysosome biogenesis begins with ATG5-ATG12-ATG16L1 forming phagophores, followed by protein recruitment requiring ATG7 and ATG10 catalytic activity [14]. Our previous research detected autophagy and autolysosomes during cochlear KO-support cell regression, with ATP stored in lysosomes [15]. In this study, a mouse model with specific autophagy knockout in KO supporting cells showed that autophagy impairment led to adult hearing loss. *Atg5*-related autophagy impairment disrupted the autophagy–lysosome pathway, reducing the lysosomal marker LAMP1, suggesting a decline in lysosome biogenesis as the cause.

*Foxa3*, a member of the fork-head box (FOX) family with a conserved DNA-binding domain, is involved in many physiological processes as part of the FOX transcription factor family [16,17]. The *Foxa3* gene is key in developmental regulation [18]. FOXA3 activates target gene transcription and regulates all autophagy–lysosome pathway stages [19,20]. Studies show strong FOXA3 expression in early development, crucial for cell and tissue homeostasis [21]. This study used conditional knockout mouse genetics, transcriptomics, and auditory electrophysiology to verify FOXA3’s role in regulating autophagy–lysosome biogenesis during mouse cochlear KO supporting cell degeneration. FOXA3 was robustly expressed in early KO supporting cells and declined in parallel with physiological autophagy flux. In vitro knockdown of *Foxa3* and *Atg5* genes reduced LAMP1 expression. This study identifies the ATG5-FOXA3 axis in lysosomal biogenesis, providing insights into mechanisms of congenital sensorineural hearing loss.

## 2. Materials and Methods

### 2.1. Animals and Genotyping

*Atg5flox* mice (Cat#1277186, Shanghai Biomodel Organism, Shanghai, China) were crossed with *Sox2-*creER mice (Cat#017593, The Jackson Laboratory, Bar Harbor, ME, USA) to cochlea-supporting cell knockout. Both male and female mice were used without gender restriction, with n = 3 or 6 (mice or cochleae) per group for experiments. Tamoxifen dissolved in corn oil (50 μL at 50 mg/mL, corresponding to 100 mg/kg body weight) was injected into pups once daily from p3 through p5 to induce *Atg5^flox/flox^*; *Sox2Cre+* mice; genotyping for both *Sox2-*creER and *Atg5*flox was subsequently performed. Genotyping primers are as follows: 5′-TTCAGTGTACCCTGTGTATTGG-3′ (primer 1, *Atg5*), 5′-GGGAAACAGTTGTGTTCTTTGT-3′ (primer 2, *Atg5*); 5′-GCGGTCTGGCAGTAAAAACTATC-3′ (primer 1, *Sox2*-creER), 5′-GTGAAACAGCATTGCTGTCACTT-3′ (primer 2, *Sox2*-creER), 5′-CTAGGCCACAGAATTGAAAGATCT-3′ (primer 3, *Sox2*-creER), and 5′-GTAGGTGGAAATTCTAGCATCATCC-3′ (primer 4, *Sox2*-creER). All experimental procedures conformed to the guidelines of the Institutional Animal Care and Use Committee of Shanghai Children’s Hospital, School of Medicine, Shanghai Jiao Tong University. Approval number: SHCH-IACUC-2025-XMSB-14. Mice were anesthetized with ketamine (100 mg/kg) and xylazine (10 mg/kg).

### 2.2. Tissue Preparation and Immunostaining

Frozen sections: Cochleae (n = 3 cochleae from 3 mice/group) were carefully dissected, fixed in 4% PFA for 24 h at 4 °C, decalcified in 10% EDTA for 1 day, and dehydrated through a graded sucrose series before embedding in OCT compound. Frozen sections (8 µm) were cut and stored at −80 °C. After permeabilization and blocking, sections were incubated with primary antibodies FOXA3 (Invitrogen, Carlsbad, CA, USA, #PA5-106980, 1:200), Myosin7a (Santa Cruz, CA, USA, #sc-74516, 1:200) overnight at 4 °C, washed, and then incubated with appropriate secondary antibodies for 2 h at 37 °C. Nuclei were stained with DAPI, sections were washed in 1xPBS and mounted with antifade medium, and images were acquired on a Leica SP8 confocal microscope at 0.5 µm z-steps and processed with Image J (v2.1.0). For quantification, fluorescence intensity was measured in regions of interest using Image J software (v2.1.0), with background subtraction and blinded analysis.

Cochlear whole-mount staining: Cochleae (n = 3 cochleae from 3 mice/group) from 2-month-old mice were dissected and fixed in 4% PFA for 24 h at 4 °C. After decalcification in 10% EDTA for 1 day, the basilar membrane was carefully micro-dissected from the cochlear duct, and the tectorial membrane was gently removed. Samples were stained with Alexa Fluor 594-phalloidin (1:200, Sigma, MO, USA, #51927) for 0.5 h at room temperature to visualize F-actin in hair cell stereocilia. After washing in PBS, whole-mount preparations were imaged using a Leica SP8 confocal microscope (40× objective). OHCs were counted in three 200 µm segments per cochlear turn (apical, middle, basal), and survival rates were calculated relative to control mice. Only OHCs with intact cuticular plates and stereocilia bundles were counted as surviving.

### 2.3. RNA-Seq and Bioinformatics

Total RNA was extracted from P3 cochlear basilar membrane dissected in cold PBS. A total of 6 cochleae without vestibular parts from 3 mice in different experimental group groups were pooled for RNA extraction. TRIZOL reagent (BBI, Shanghai, China, #B610409) was used to extract total RNA following the manufacturer’s instructions. RNA purity and quantification were evaluated using the Nanodrop 2000 spectrophotometer (Thermos Scientific, Waltham, MA, USA). RNA integrity was assessed using the Agilent 2100 Bioanalyzer (Agilent Technologies, Santa Clara, CA, USA). Then, the libraries were constructed using VAHTS Universal V6 RNA-seq Library Prep Kit (Vazyme Biotech, Nanjing, China), then sequenced on an Illumina Novaseq 6000 platform (San Diego, CA, USA), and 150 bp paired-end reads were generated. PCA analysis was performed using R (https://www.r-project.org) to evaluate the biological duplication of samples. Differential expression analysis was performed using the DESeq2. Q value < 0.05 and foldchange > 2 or foldchange < 0.5 was set as the threshold for significantly differential expression genes (DEGs). Gene set enrichment analysis (GSEA) was performed using GSEA software (v4.1.0) with 1000 permutations. Multiple testing correction was applied using the Benjamini–Hochberg method.

### 2.4. Auditory Physiology

Prior to ABR recording, all mice underwent otoscopic examination to exclude conductive pathology (tympanic membrane perforation, effusion, or debris). Only mice with intact middle ear structures were included. ABR (n = 6 mice/group) was recorded in a sound-attenuated chamber (TDT RZ6, Alachua, FL, USA). Needle electrodes were inserted at the vertex, ventrolateral to the left pinna, and the right forelimb to serve as active, reference, and ground electrodes, respectively. Stimuli were delivered via a free-field speaker (MF1, Tucker-Davis Technologies) positioned 10 cm from the left ear. Tone-burst stimuli (3 ms duration, 1 ms rise/fall, 21.3/s repetition rate, 4–32 kHz frequency range) were delivered. Click stimuli (0.1 ms rectangular pulses) were also presented. The sampling rate was 50 kHz with bandpass filtering (0.3–3 kHz) and 512 averaged responses. Threshold was defined as the lowest intensity producing a visible Wave I.

### 2.5. KO Cells Culture and Foxa3-KD Lentiviral Vector Construction and Transfection

P3 *Atg5flox/flox*; *Sox2Cre+* and *Atg5flox/flox*; *Sox2Cre*− mice were selected, rapidly decapitated under anesthesia, and the temporal bones were dissected. KO-supporting cells were located medial to the inner hair cells. Segments of the basilar membrane containing the KO region were carefully dissected using fine needles and transferred to a centrifuge tube containing 0.25% trypsin-EDTA (Gibco, Grand Island, NY, USA, #25200072) for enzymatic digestion at 37 °C for 5 min. The suspension was gently triturated every 2 min during digestion. Digestion was terminated by adding DMEM/F12 medium (Gibco, Grand Island, NY, USA, #11320033) supplemented with 10% FBS (Gibco, Grand Island, NY, USA, #A5256701) and 1% penicillin (Sangon, Shanghai, China, #A430123). The cells were pelleted by centrifugation at 1000 rpm for 5 min, the supernatant was discarded, and the cells were resuspended in fresh culture medium. Cells were plated at a density of 1 × 10^5^ cells/mL in 24-well plates and cultured in a humidified incubator at 37 °C with 5% CO_2_. After 24 h, the KO cells had adhered, and they were transduced with a lentiviral vector. Lentiviral shRNA vector targeting Foxa3 (pLKO.1-U6-shFoxa3-CMV-EGFP-Puro) was constructed and packaged by Obio Technology (Shanghai, China); viral titer: 10^9^ TU/mL. Cells were infected for 48 h and then selected with puromycin for 7 days to establish a stable Foxa3-KD cells.

### 2.6. Quantitative Real-Time PCR (qRT-PCR)

Total RNA was extracted from cultured KO cells or cochlear basilar membranes using TRIZOL reagent (BBI, Shanghai, China, #B610409) and reverse-transcribed using Prime Script RT Master Mix (TIANGEN, Beijing, China, #KR123) according to the manufacturer’s protocol. Primers used: Foxa3 (forward: 5′-AGCAGCGAGTACATCAAACAG-3′, reverse: 5′-GGTGACAGCAGCTGTAGTTCA-3′). Atg5 (forward: 5′-CCTTTCATTCAGAAGCTGTTTCC-3′, reverse: 5′-CTGTCCATTGATGTGCTTCATC-3′), Map1lc3b (forward: 5′-GATGTCCGACTTATTCGAGAGC-3′, reverse: 5′-TTGGGAGGCATAGACCATGTGA-3′), Lamp1 (forward: 5′-TGGCAGCTGTCATGTTTCAG-3′, reverse: 5′-CCAGGATGGTGGAAGAGTGC-3′), and Lamp2 (forward: 5′-CTGGTGGACAGTGATGTGGA-3′, reverse: 5′-GAGGTTGGTGCTGTCTGTGA-3′), and Lamp5 (F: 5′-GCTGCTGTGGTGGTGTTTGT-3′, R: 5′-AGGGTGGTGGTGGTGGTAGA-3′). Gapdh served as internal control. Relative expression was calculated using the 2^−ΔΔCt^ method. Each sample was analyzed in triplicate, and data are presented as mean ± SD, with n = 3 mice /group.

### 2.7. Statistics

Data are presented as mean ± SD. Normality was assessed with Shapiro–Wilk test. Comparisons between two groups were performed using unpaired two-tailed Student’s *t*-test. Multiple group comparisons were analyzed by one-way ANOVA with Dunnett’s post hoc test. ABR threshold data across frequencies were analyzed by two-way ANOVA with Bonferroni correction. RNA-seq analysis used DESeq2 with Benjamini–Hochberg correction. A *p* value < 0.05 was considered significant. Exact biological replicate numbers are indicated in figure legends. Analyses were conducted using GraphPad Prism 9.

## 3. Results

### 3.1. Developmental Autophagy in KO-Supporting Cells Is Required for Auditory Function and Outer Hair Cell Survival

**Figure 1:**
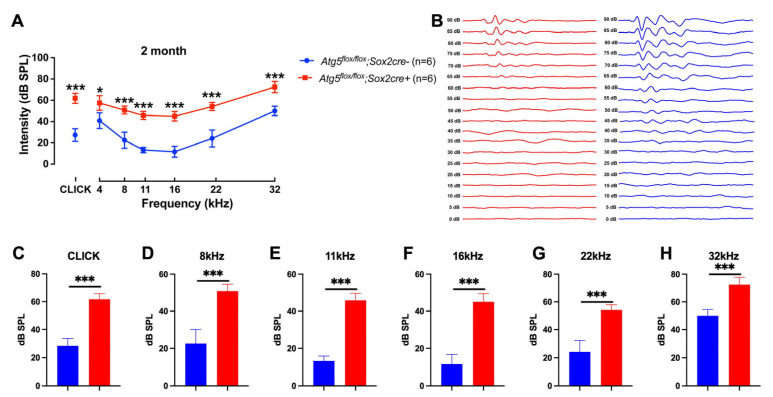
Conditional *Atg5* deletion in *SOX2^+^* progenitors cause moderate-to-severe sensorineural hearing loss in 2-month-old mice. (**A**) ABR audiograms of 2-month-old mice. (**B**) Representative ABR waveforms were acquired using click stimuli to display characteristic Wave I–V morphology. (**C**–**H**) ABR thresholds at 8–32 kHz and click show a 25- 40 dB elevation in 2-month-old *Atg5flox/flox*; *Sox2Cre+* mice (Red) versus controls (*Atg5flox/flox*; *Sox2Cre−* mice, blue). Data are mean ± SD, two-way ANOVA with Bonferroni correction. (* *p* < 0.05, *** *p* < 0.001, n = 6).

To determine whether ATG5-dependent autophagy within cochlea-supporting cells is necessary for hearing, we induced conditional Atg5 deletion by tamoxifen injection in *Atg5flox/flox*; *Sox2Cre+* pups at P3–P5 and assessed auditory function at 2 months of age (P60). ABR recordings revealed moderate-to-severe sensorineural hearing loss in mutant mice, manifesting as a 25–40 dB elevation in thresholds across 4–32 kHz (Figure 1A). Mean click thresholds increased from 20 ± 3 dB SPL in *Atg5flox/flox*; *Sox2Cre−* mice to 60 ± 4 dB SPL in *Atg5flox/flox*; *Sox2Cre+* mice (*p* < 0.001, n = 6 per group). Representative ABR waveforms acquired using click stimuli displayed the characteristic Wave I–V morphology, with clear threshold elevation and preserved peak latencies in knockout mice (Figure 1B).

**Figure 2:**
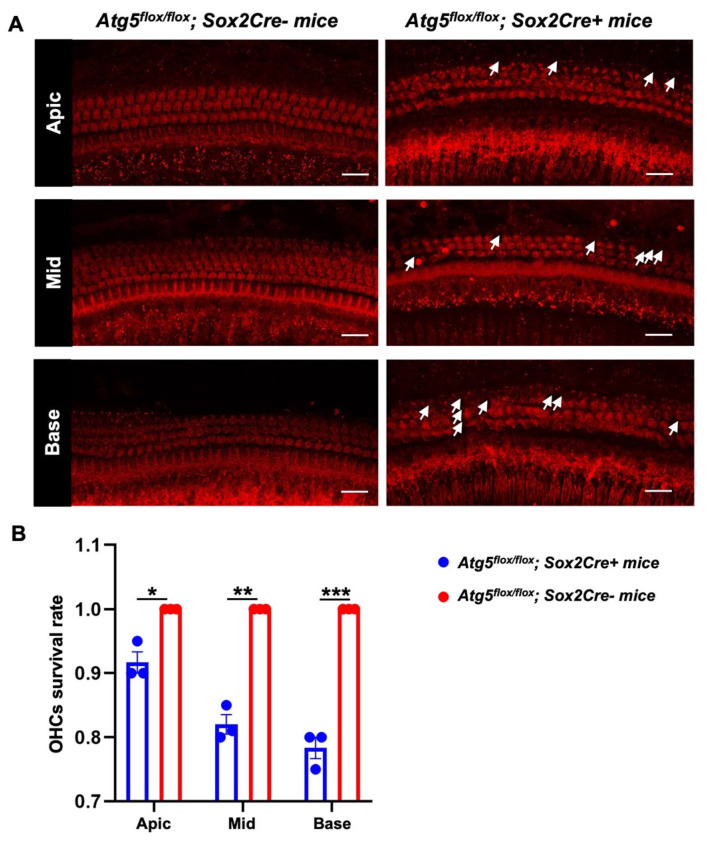
Conditional *Atg5* deletion causes outer hair cell loss. (**A**) Representative phalloidin staining (red) of cochlear whole mounts from 2-month-old *Atg5flox/flox*; *Sox2Cre+* mice versus *Atg5flox/flox*; *Sox2Cre−* mice showing outer hair cell morphology in the apical (Apic), middle (Mid), and basal (Base) turns. White arrows indicate missing outer hair cells. Scale bar: 25 μm. (**B**) Quantification of outer hair cell (OHCs) survival rates across the apical, middle, and basal turns showing significant reduction in *Atg5flox/flox*; *Sox2Cre+* mice (blue) versus controls (*Atg5flox/flox*; *Sox2Cre−* mice, red). Data are mean ± SD. (* *p* < 0.05, ** *p* < 0.01, *** *p* < 0.001, n = 3 cochlea samples).

To ascertain the cellular basis of this hearing impairment and exclude conductive pathology, we examined outer hair cell (OHC) integrity via phalloidin staining of cochlear whole mounts. While *Atg5flox/flox*; *Sox2Cre−* mice displayed robust, orderly rows of OHCs with intact stereocilia bundles throughout all cochlear turns, mutant mice exhibited significant OHC loss and architectural disarray, particularly in the basal and middle regions (Figure 2A). Inner hair cells (IHCs) showed no obvious loss in either genotype. Quantitative analysis confirmed reduced OHC survival rates in *Atg5*-deficient cochleae relative to *Atg5flox/flox*; *Sox2Cre−* (Figure 2B). Collectively, these findings demonstrate that developmental autophagy deficiency in KO-supporting cells compromises auditory function through progressive OHC degeneration, confirming the sensorineural hearing loss.

### 3.2. Transcriptomic Profiling Identifies FOXA3 as an Autophagy-Sensitive Transcription Factor in the Developing Cochlea

**Figure 3:**
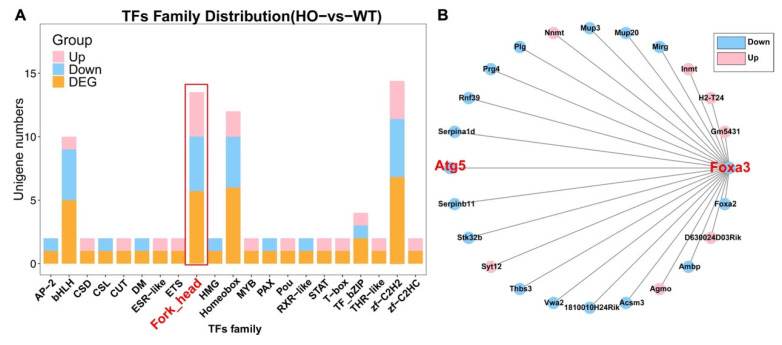
Transcriptomic analysis identifies downregulation of fork-head family transcription factors in *Atg5*-deficient cochleae. (**A**) Transcription factor family distribution showing enrichment of fork-head family genes (red box) among down-regulated genes in *Atg5flox/flox*; *Sox2Cre+* mice (HO) versus *Atg5flox/flox*; *Sox2Cre−* mice (WT). (**B**) Gene association network displaying Foxa3 and Atg5 (red) and their correlated genes. Blue indicates down-regulated genes; pink indicates up-regulated genes.

To uncover the molecular mechanisms linking ATG5 deficiency to hearing impairment, we performed bulk RNA-seq on P3 basilar membranes (n = 2 cochleae per replicate, 3 biological replicates per genotype). Transcription-factor family annotation identified the fork-head (FOX) family as significantly enriched among down-regulated transcription factors (Figure 3A). Gene association network analysis further positioned Foxa3 and Atg5 as central hub nodes within the down-regulated autophagy–lysosome gene module (Figure 3B).

**Figure 4:**
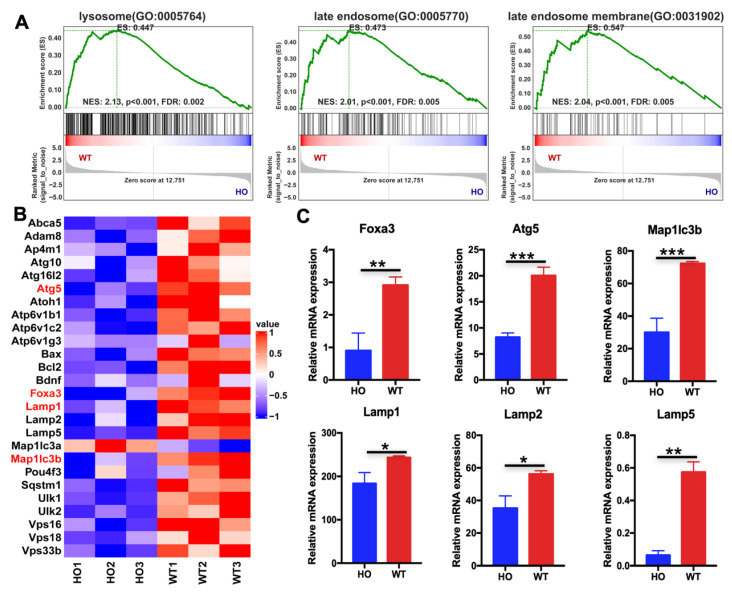
RNA-seq analysis reveals downregulation of lysosomal and autophagy genes in *Atg5*-deficient cochlea. (**A**) Gene set enrichment analysis (GSEA) showing significant down-regulation of lysosome (GO:0005764), late endosome (GO:0005770), and late endosome membrane (GO:0031902) gene sets in P3 *Atg5^flox/flox^*; *Sox2Cre+* (HO) compared to *Atg5^flox/flox^*; *Sox2Cre−* (WT) basilar membranes. NES, normalized enrichment score; FDR, false discovery rate. (**B**) Heat-map visualization of differentially expressed autophagy and lysosomal genes across biological replicates (HO1–3: *Atg5^flox/flox^*; *Sox2Cre+*; WT1–3: *Atg5^flox/flox^*; *Sox2Cre−*). Color scale indicates row-normalized expression values (Z-score). (**C**) Quantitative RT-PCR validation of selected down-regulated genes (Foxa3, Atg5, Map1lc3b, Lamp1, Lamp2, Lamp5) in HO versus WT cochleae. Data are mean ± SD. * *p* < 0.05, ** *p* < 0.01, *** *p* < 0.001 (Student’s *t*-test), n = 3.

Gene-set enrichment analysis (GSEA) revealed significant down-regulation of lysosomal (GO:0005764), late-endosome (GO:0005770), and late-endosome membrane (GO:0031902) gene signatures in *Atg5flox/flox*; *Sox2Cre+* mice cochleae compared to controls, with normalized enrichment scores (NES) ranging from 2.01 to 2.13 and false discovery rates (FDR) < 0.01 (Figure 4A). Detailed examination of differentially expressed genes confirmed broad suppression of the lysosomal and autophagy network in mutant cochleae. Hierarchical clustering heatmaps revealed coordinate down-regulation of core autophagy machinery (Atg5, Atg16l1, Map1lc3a/b), lysosomal membrane markers (Lamp1, Lamp2, Lamp5), and the transcription factor Foxa3 (Figure 4B). Quantitative RT-PCR validation confirmed significant reductions in Foxa3, Atg5, Map1lc3b, and lysosomal markers Lamp1, Lamp2, and Lamp5 in *Atg5*-deficient tissue compared to WT (Figure 4C). These transcriptomic findings indicate that ATG5 loss precipitates transcriptional collapse of the lysosomal gene network and specifically implicates FOXA3 as a potential autophagy-regulated controller of lysosomal biogenesis in the developing cochlea.

### 3.3. FOXA3 Exhibits Developmental Stage-Specific Expression with Progressive Restriction to KO-Supporting Cells

**Figure 5:**
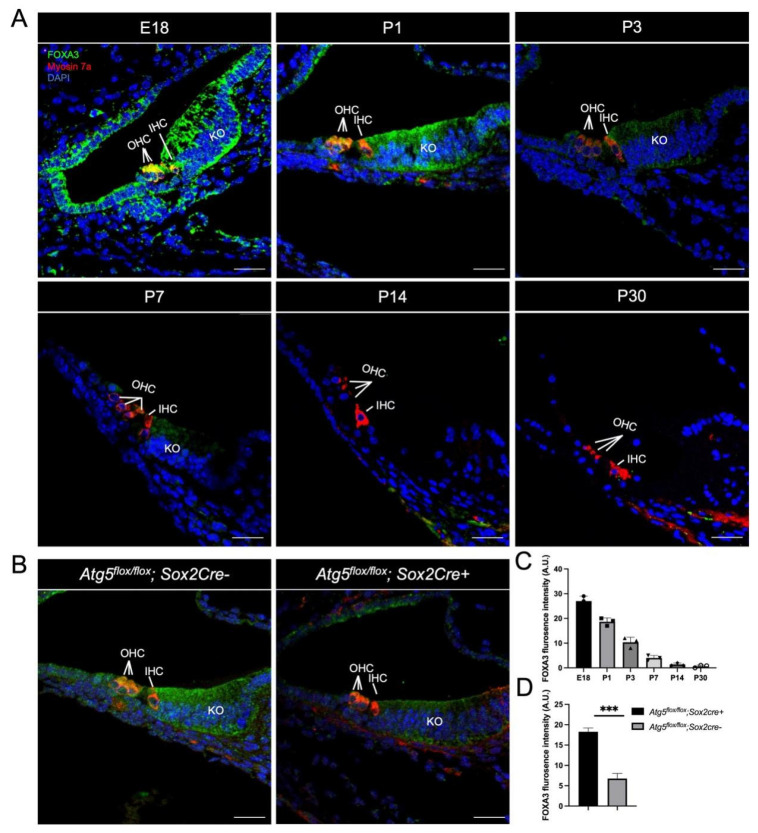
FOXA3 expression mirrors the autophagy time-course and becomes progressively restricted to KO-supporting cells. (**A**) Immunofluorescence showing spatiotemporal expression of FOXA3 in the developing cochlea; FOXA3 is enriched in *SOX2^+^* KO-cell domains, green: FOXA3; red: Myosin7a (hair-cell marker); blue: DAPI (nuclei). (**B**) Representative images illustrating that *Atg5*-KD markedly reduces FOXA3 levels in KO cells, green: FOXA3; red: Myosin7a (hair-cell marker); blue: DAPI (nuclei). (**C**) Quantitative analysis of FOXA3 fluorescence intensity expression declines progressively after birth. (**D**) Quantification confirms that *Atg5*-KD significantly lowers FOXA3 fluorescence intensity in KO cells. Datas are mean ± SD. (*** *p* < 0.01, n = 3 cochlea samples). Scale bar: 25 µm.

Having identified Foxa3 as a downregulated transcription factor in our transcriptomic analysis, we next characterized its spatiotemporal protein expression during cochlear development. Immunofluorescence staining revealed dynamic changes in FOXA3 localization from embryonic day 18 (E18) to postnatal day 30 (P30). At E18, strong FOXA3 immunoreactivity was detectable in both KO cells and hair cells, indicating transient expression throughout the developing sensory epithelium (Figure 5A). From P1 to P3, FOXA3 remained present in both cell types, though progressively enriched in KO-supporting cells relative to hair cells (Figure 5A). FOXA3 expression became completely undetectable in hair cells, persisting exclusively in KO cells at P7 (Figure 5A). Quantitative analysis revealed that signal intensity in KO cells declined progressively thereafter, dropping approximately 6-fold by P7 compared to P1, and becoming barely detectable by P14 (Figure 5C). At E18 and P1, FOXA3 immunoreactivity was observed in both nuclear and cytoplasmic compartments of KO-supporting cells, with relatively stronger signal intensity in the cytoplasm/perinuclear region compared to nuclei (Figure 5A). This dual-compartment presence persisted through P3, a stage characterized by active developmental remodeling. By P7, FOXA3 signal became predominantly cytoplasmic in KO cells, with reduced or minimal nuclear detection, while expression in hair cells was no longer observable (Figure 5A).

To determine whether ATG5-dependent autophagy maintains FOXA3 protein levels, we examined *Atg5* deficient cochleae at P1. Representative images revealed that ATG5 deficiency selectively reduced FOXA3 levels in KO cells, which normally exhibit strong expression at this stage, while sparing the minimal residual expression in neighboring hair cells. (Figure 5B). Quantitative analysis confirmed a 62.4% decrease in FOXA3 fluorescence intensity in *Atg5flox/flox*; *Sox2Cre+* KO cells compared to *Atg5flox/flox*; *Sox2Cre−* (Figure 5D). These results establish that FOXA3 is an autophagy-regulated transcription factor whose expression becomes progressively restricted to KO supporting cells during the critical developmental window.

### 3.4. FOXA3 and ATG5 Are Required for Lysosomal Maintenance in KO Supporting Cells

**Figure 6:**
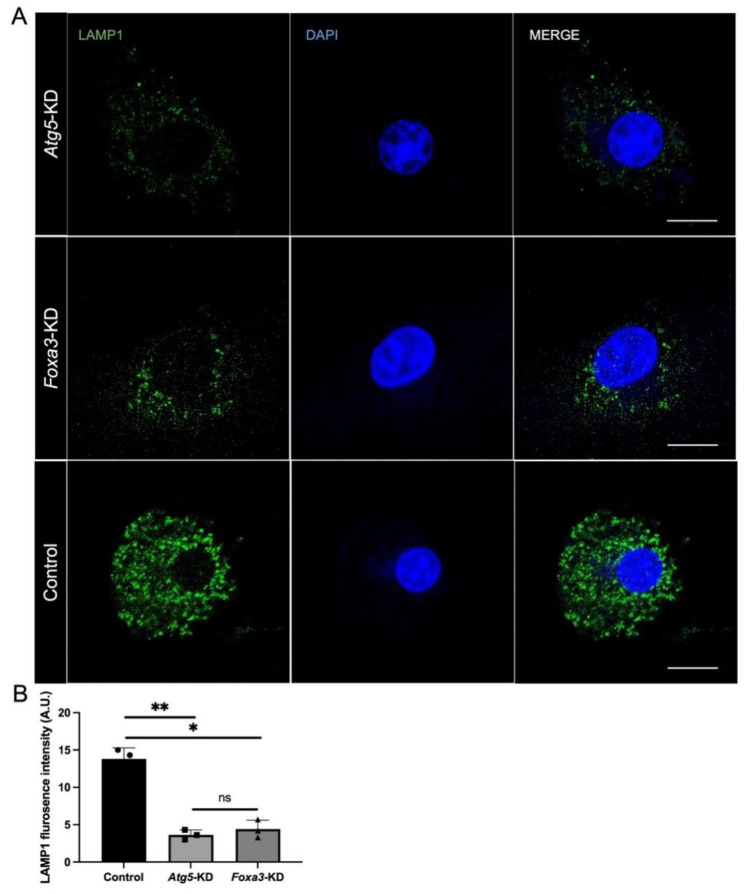
Deficiency of either *Atg5* or *Foxa3* reduces lysosomal abundance in KO cells. (**A**) LAMP1 immunofluorescence in KO cells with *Atg5* deficiency (*Atg5*-KD) or *Foxa3* knockdown (*Foxa3*-KD), green: LAMP1 (lysosomes); blue: DAPI (nuclei). (**B**) Quantification of lysosome abundance in KO cells; ns *p* > 0.05, * *p* < 0.05, ** *p <* 0.01. Scale bar: 5 µm.

To investigate whether the ATG5-FOXA3 axis functionally contributes to lysosomal biogenesis, we established primary cultures of P3 KO supporting cells and performed independent knockdown experiments. Foxa3 knockdown was confirmed by qRT-PCR (Figure S1C). Quantification of LAMP1 positive puncta revealed that Atg5 knockdown resulted in a 73.7% decrease in lysosomal abundance compared to scrambled controls (Figure 6A,B). Similarly, Foxa3 knockdown produced a comparable 68.1% reduction in LAMP1 levels (Figure 6A,B). These findings demonstrate that both ATG5 and FOXA3 contribute to lysosomal maintenance in KO supporting cells. The similar magnitude of lysosomal depletion observed upon disruption of either factor supports a functional relationship within the ATG5-FOXA3 axis, though additional rescue experiments are needed to establish causal sufficiency.

## 4. Discussion

### 4.1. Early Auditory Development: Normal Degeneration of KO Supporting Cells Depends on Autophagy

Our previous study showed that a comparison of the expression trends of autophagy- and apoptosis-related proteins revealed that the expression peak of autophagy-related proteins occurred at P1, earlier than the peak of apoptosis-related proteins (P7–10) [9]. The current study extends these findings by demonstrating that conditional deletion of *Atg5* in KO supporting cells results in moderate-to-severe sensorineural hearing loss at 2 months of age, accompanied by significant OHC loss (Figure 2). These data support a model wherein developmental autophagy in KO cells is required for the programmed degeneration of these transient supporting cells and for subsequent long-term maintenance of the auditory sensory epithelium. We clarify that KO degeneration represents a programmed cell death process essential for cochlear maturation, and that impairment of this process leads to permanent structural defects evidenced by OHC loss at P60, rather than merely developmental delay.

### 4.2. Autophagy–Lysosome Biogenesis Is Crucial for Auditory Development Maturity

Lysosome function is intricately linked to the development and maturation of the auditory and nervous systems [21,22]. The autophagosome–lysosome pathway is the sole route for complete organelle degradation, facilitating the rapid degradation of organelles and cytoplasmic components in developing, especially regressing, cells to achieve cell volume reduction [23,24]. Additionally, autophagy can maintain an appropriate high ATP concentration to promote cell apoptosis [25]. Therefore, autophagy and apoptosis jointly regulate the orderly regression of KO-supporting cells. Autophagy–lysosome pathway abnormalities, including impaired lysosome biogenesis, defective autophagosome maturation, abnormal autophagosome–lysosome fusion, and reduced lysosomal degradation capacity, are major drivers of auditory and nervous system diseases [26,27].

Most lysosomal storage diseases stem from synthesis disorders or reduced activity of lysosomal hydrolases [28,29]. Examples like Gaucher disease (GBA gene mutation), Fabry disease (ɑ-galactosidase A gene mutation), Pompe disease (acid ɑ-glucosidase gene mutation), and mannosidosis (mannosidase gene mutation) all present with audiological abnormalities [30,31,32]. Additionally, abnormal lysosome function can directly cause auditory impairment. Mutations in the ATP6V1B2 protein, crucial for lysosomal acidic environment and hydrolase activity and highly expressed in mouse inner ear hair cells and spiral neurons, lead to hearing loss onychodystrophy syndrome [33,34]. Mutations in ATP6V1B1 and ATP6V0A4, lysosomal proton pump genes, result in syndromic deafness with distal renal tubular acidosis [35,36]. These findings highlight the close connection between lysosome function and the auditory conduction pathway, yet the impact on cochlear supporting cell development and maturation remain unreported.

Our previous studies have shown that autophagy and autolysosomes exist during the degeneration of cochlear KO-supporting cells, and ATP is stored in lysosomes [15]. Our transcriptomic and cellular analyses reveal that ATG5 deficiency leads to downregulation of the entire lysosomal gene network, including v-ATPase subunits and LAMP proteins (Figure 3 and Figure 4). This is consistent with reports linking lysosomal storage disorders and mutations in lysosomal proton pumps (e.g., ATP6V1B2) to hearing impairment. However, our study specifically implicates defective autophagy–lysosomal degradation during the degeneration of KO cells as a mechanism for secondary OHC loss. In the future, we will analyze the functional changes in lysosomes and lysosomal proteases during the regression of mouse cochlear KO-supporting cells to clarify the mechanism by which autophagy–lysosome biogenesis promotes auditory development maturity.

### 4.3. FOXA3 as a Potential Autophagy-Sensitive Regulator

FOXA3 belongs to the fork-head box (FOX) family of transcription factors, which are evolutionarily conserved regulators involved in diverse biological processes including development, metabolism, and cell fate determination [37,38,39]. FOX transcription factors are also known to regulate multiple signaling pathways and transcriptional networks, including Wnt/β-catenin-dependent gene transcription, highlighting their broad regulatory roles in developmental processes [40].

Our findings position FOXA3 as a transcription factor whose expression and function are linked to ATG5-dependent autophagy in KO supporting cells. The progressive restriction of FOXA3 expression from a broad sensory epithelial pattern at E18 to KO specific enrichment by P7 (Figure 5) parallels the autophagy time-course during KO degeneration and supports a role in this developmental cell death process. Predominantly cytoplasmic localization at this stage may reflect developmental stage-specific nucleocytoplasmic shuttling. The observation that *Atg5* deficiency reduces FOXA3 protein levels by 62.4% (Figure 5D), combined with the similar lysosomal phenotypes observed upon knockdown of either factor (Figure 6), suggests that FOXA3 may act downstream of ATG5 to maintain lysosomal biogenesis required for orderly KO degeneration. Evidence from inner ear studies further supports a link between FOX family transcription factors and autophagy-related mechanisms in auditory cells. For example, FOXG1 has been shown to regulate autophagy pathways to promote hair cell survival and modulate inner ear development and cellular homeostasis [41,42]. In addition, manipulation of *Foxg1* expression in cochlear supporting cells can influence cell fate transitions and hair cell regeneration potential [43]. FOX family transcription factors have also been reported to regulate inflammatory sensitivity and stress responses in aging hair cells through autophagy-related pathways [44].

However, we emphasize that current data support a regulatory association rather than a linear mechanistic axis; alternative explanations, including parallel pathways or feedback loops, cannot be excluded without epistasis analysis and rescue experiments. FOXA3 knockdown in cultured KO cells recapitulates the lysosomal deficits observed in *Atg5*-deficient cells (Figure 6), supporting cell-autonomous roles for both factors in regulating lysosomal function during degeneration. Future ChIP-seq, rescue experiments, and autophagic flux measurements (LC3-II/p62) are needed to validate this regulatory axis and clarify lysosomal mechanisms.

In summary, our data suggest that ATG5-dependent autophagy is associated with the maintenance of FOXA3 expression and lysosomal integrity in KO supporting cells during early postnatal development. Disruption of this pathway correlates with outer hair cell degeneration and sensorineural hearing loss in adult mice. These data show association, not causation. Subsequent rescue experiments and ChIP-seq analysis will be required to define the regulatory hierarchy.

## Data Availability

The original contributions presented in this study are included in the article/Supplementary Material. Further inquiries can be directed to the corresponding authors.

## Supplementary Materials

The following supporting information can be downloaded at https://www.mdpi.com/article/10.3390/biomedicines14040802/s1. Figure S1. Validation of ATG5 deficiency in conditional knockout mice and knockdown efficiency in primary KO cells.
