# Supplementary material for: ATG5-FOXA3 Axis Contributes to Lysosomal Biogenesis and Auditory Function in Kölliker’s Organ
Source: Biomedicines. 2026 Apr 1;14(4):802. doi: 10.3390/biomedicines14040802 (PMC13113778; doi:10.3390/biomedicines14040802)
Supplement: Supplementary file 1 [file biomedicines-14-00802-s001.zip › biomedicines-4131517-supplementary.pdf]

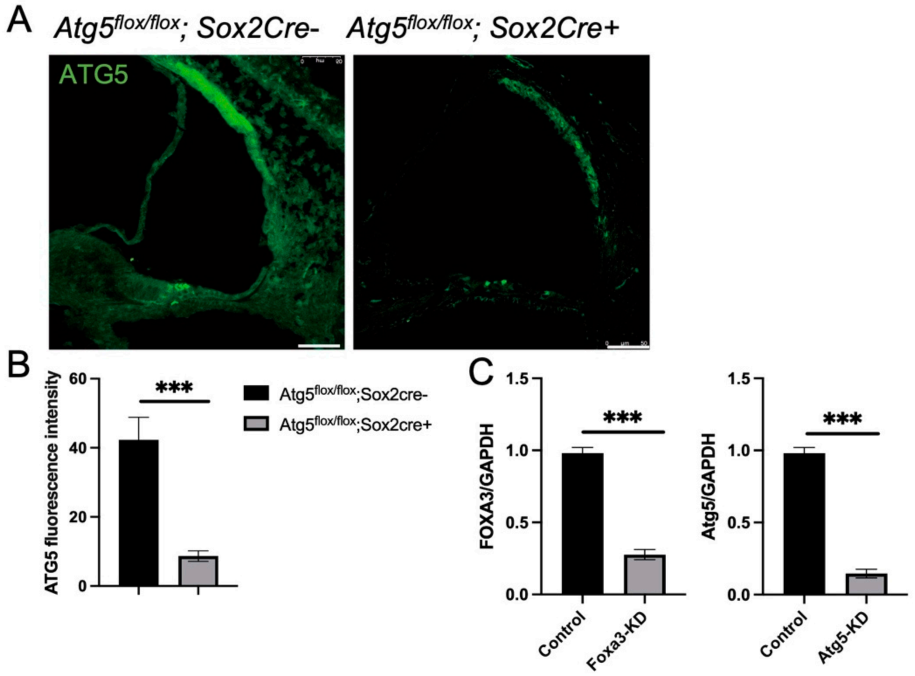

Figure S1. Validation of ATG5 deficiency in conditional knockout mice and knockdown efficiency in primary KO cells. (A) Representative immunofluorescence images showing ATG5 expression (green) in the cochlea of *Atg5$^{flox/flox}$; Sox2Cre*- and *Atg5$^{flox/flox}$; Sox2Cre+* mice at P3. Scale bar: 50 µm. (B) Quantification of ATG5 fluorescence intensity in the KO cell region (n = 3 biological replicates per group). (C) qRT-PCR validation of knockdown efficiency in primary KO cell cultures. Left: Foxa3 mRNA levels in control versus Foxa3-KD cells; Right: Atg5 mRNA levels in control versus Atg5-KD cells (n = 3 biological replicates per group). Data are mean ± SD. (****P < 0.001*, Student's t-test).
